# *catena*-Poly[(aqua­dimethano­lzinc)-μ-furan-2,5-dicarboxyl­ato-κ^3^ *O* ^2^:*O* ^2^,*O* ^2′^]

**DOI:** 10.1107/S1600536812014936

**Published:** 2012-04-13

**Authors:** Ya-Feng Li, Yue Gao, Yue Xu, Xiao-Lin Qin, Wen-Yuan Gao

**Affiliations:** a School of Chemical Engineering, Changchun University of Technology, Changchun 130012, People’s Republic of China

## Abstract

In the crystal structure of the title compound, [Zn(C_6_H_2_O_5_)(CH_3_OH)_2_(H_2_O)]_*n*_, an infinite chain is formed along the *b* axis by linking of the Zn(OH_2_)(CH_3_OH)_2_ unit with one carboxyl­ate group of the furan-2,5-dicarboxyl­ate ligand. The Zn^II^ ion is in a distorted octa­hedral environment with one weak coordination [Zn—O_carboxyl­ate_ = 2.565 (3) Å] and two meth­anol mol­ecules located in axial positions. In the chain, O_water_—H⋯O hydrogen bonds are present, while adjacent chains are linked by O_methanol_—H⋯O hydrogen bonds into a layer parallel to (10-2).

## Related literature

For applications and structures of metal-organic framework materials, see: Chui *et al.* (1999 ▶); Corma *et al.* (2010 ▶); Ferey (2008 ▶); Li *et al.* (1999 ▶, 2012*a* ▶,*b* ▶); Ma *et al.* (2009 ▶); Murray *et al.* (2009 ▶); Tranchemontagne *et al.* (2009 ▶).
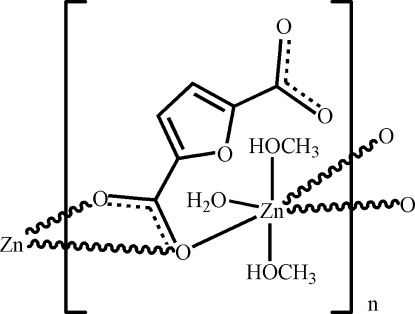


## Experimental

### 

#### Crystal data

[Zn(C_6_H_2_O_5_)(CH_4_O)_2_(H_2_O)]
*M* *_r_* = 301.57
Monoclinic, 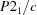

*a* = 10.077 (2) Å
*b* = 8.1235 (16) Å
*c* = 17.101 (3) Å
β = 124.86 (3)°
*V* = 1148.7 (6) Å^3^
*Z* = 4
Mo *K*α radiation
μ = 2.17 mm^−1^
*T* = 293 K
0.10 × 0.10 × 0.10 mm

#### Data collection

Rigaku R-AXIS RAPID diffractometer
Absorption correction: multi-scan (*ABSCOR*; Higashi, 1995 ▶) *T*_min_ = 0.813, *T*_max_ = 0.813
10467 measured reflections
2605 independent reflections
1593 reflections with *I* > 2σ(*I*)
*R*_int_ = 0.119

#### Refinement

*R*[*F* ^2^ > 2σ(*F* ^2^)] = 0.062
*wR*(*F* ^2^) = 0.142
*S* = 1.01
2605 reflections
168 parameters
5 restraints
H atoms treated by a mixture of independent and constrained refinement
Δρ_max_ = 0.61 e Å^−3^
Δρ_min_ = −0.71 e Å^−3^

### 

Data collection: *PROCESS-AUTO* (Rigaku, 1998 ▶); cell refinement: *PROCESS-AUTO*; data reduction: *CrystalStructure* (Rigaku/MSC, 2002 ▶); program(s) used to solve structure: *SHELXS97* (Sheldrick, 2008 ▶); program(s) used to refine structure: *SHELXL97* (Sheldrick, 2008 ▶); molecular graphics: *DIAMOND* (Brandenburg, 2000 ▶); software used to prepare material for publication: *SHELXL97*.

## Supplementary Material

Crystal structure: contains datablock(s) I, global. DOI: 10.1107/S1600536812014936/is5106sup1.cif

Structure factors: contains datablock(s) I. DOI: 10.1107/S1600536812014936/is5106Isup2.hkl

Additional supplementary materials:  crystallographic information; 3D view; checkCIF report

## Figures and Tables

**Table 1:** Hydrogen-bond geometry (Å, °)

*D*—H⋯*A*	*D*—H	H⋯*A*	*D*⋯*A*	*D*—H⋯*A*
O1*W*—H1*A*⋯O1^i^	0.83 (2)	1.92 (2)	2.745 (5)	171 (5)
O1*W*—H1*B*⋯O5^ii^	0.83 (2)	1.76 (2)	2.571 (5)	165 (5)
O7—H7⋯O4^iii^	0.82 (2)	1.86 (3)	2.639 (5)	158 (6)
O8—H8⋯O4^iv^	0.82 (2)	1.88 (3)	2.682 (5)	163 (6)

Symmetry codes: (i) 
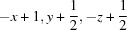
; (ii) 
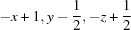
; (iii) 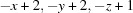; (iv) 
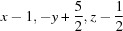
.

**Fig. 1.**
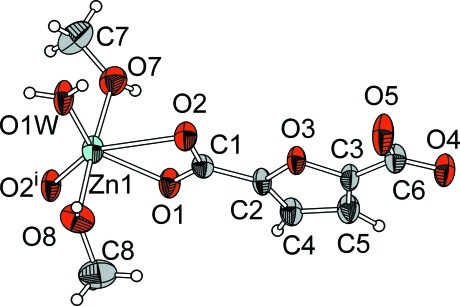
The asymmetric unit of the title compound, showing the atomic labelling scheme and displacement ellipsoids at the 50% probability level. [Symmetry code: (i) 1 - x, -1/2 + y, 1/2 - z.]

**Fig. 2.**
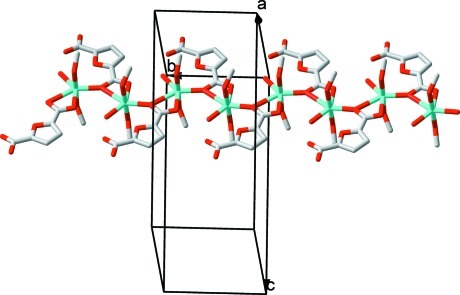
The stick plot of the title compound, displaying the infinite chain along the [010] direction formed by linking the ZnII ion with one carboxyl of furan-2,5-dicarboxylate.

**Fig. 3.**
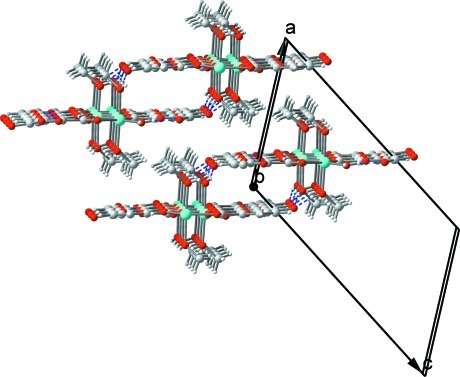
The ball-stick packing diagram of the title compound. The adjacent chains are held together by the Omethanol–H···O hydrogen bonds into layers.

## supplementary crystallographic information

### Comment

During past decades, more efforts have made to construct the metal organic framework (MOF) materials due to the potential applications including gas absorption and catalyst reactions (Ma *et al.*, 2009; Murray *et al.*, 2009; Corma *et al.*, 2010). The more attentions have been focused on the MOF based on the phenyl ring with carboxylate groups (Chui *et al.*, 1999; Li *et al.*, 1999; Ferey, 2008; Tranchemontagne *et al.*, 2009). Compared with phenyl ring with carboxylate groups, the 5-membered rings with carboxylate groups as the ligand are rarely studied. Recently, we utilize furan-2,5-dicarboxyl acid as the ligand to constructed the MOFs (Li *et al.*, 2012*a*,*b*). In this work, a novel chainlike compound, [Zn(C_6_H_2_O_5_)(H_2_O)(CH_3_OH)_2_]*_n_*, (I), is structurally determined.

The asymmetric unit of (I) contains one Zn^II^ cation, one furan-2,5-dicarboxylate anion, one water and two methanol molecules (Fig. 1). The Zn^II^ cation is coordinated by three carboxylate O atoms, one water molecule and two methanol molecules which locate at the axial positions, exhibiting a distorted octahedron. The oxygen of carboxylate [Zn—O_carboxylate_ = 2.565 (2) Å] is very weakly ligated to the Zn cation. If excluding this oxygen, the Zn^II^ ion displays a distorted triganol bipyramid geometry but the chain property may not be changed. Only one carboxyl of furan-2,5-dicarboxylate involves in the formation of infinite chain. The carboxyl shows a µ_2_:η^1^,η^2^ coordinated mode.

The Zn^II^ cations are linked by one carboxylate of furan-2,5-dicarboxylate to give rise to an infinite chain (Fig. 2). O_water_–H···O hydrogen bonds are intra-chain interactions, while O_methanol_–H···O hydrogen bonds inter-chain interactions which are responsible to link the adjacent chains into a layer parallel to the (102) plane (Fig. 3).

### Experimental

In a typically synthesized route of (I), furan-2,5-dicarboxyl acid (0.312 g, 2.0 mmol) and Zn(NO_3_)_2_^.^6H_2_O (0.592 g, 2.0 mmol) were dissolved in DMF (7.8 ml, 0.1 mol) under stirring. Then, 72 ml methanol ann N(et)_3_ (0.29 ml, 2.0 mmol) were successively added. The mixture with molar ratio of 1 (furan-2,5-dicarboxyl acid): 1 (Zn(NO_3_)_2_^.^6H_2_O): 1 (N(et)_3_) was laid under room temperature for 4 days. The colorless block product was collected as a single phase.

### Refinement

Water H atoms were located in a difference Fourier map and refined with distance restraints of O—H = 0.82 (2) Å and H···H = 1.37 (2) Å, and with *U*_iso_(H) = 1.2*U*_eq_(O). Hydroxyl H atoms were located in a difference Fourier map and refined with a restraint of O—H = 0.82 (2) Å, and with *U*_iso_(H) = 1.2*U*_eq_(O). The carbon H-atoms were placed in calculated positions [C—H (furan ring) = 0.93 Å and C—H (methyl) = 0.96 Å] and were included in the refinement in the riding-model approximation, with *U*_iso_(H) = 1.2*U*_eq_(C).

### Crystal data

**Table:**

[Zn(C_6_H_2_O_5_)(CH_4_O)_2_(H_2_O)]	*F*(000) = 616
*M**_r_* = 301.57	*D*_x_ = 1.744 Mg m^−^^3^
Monoclinic, *P*2_1_/*c*	Mo *K*α radiation, λ = 0.71073 Å
Hall symbol: -P 2ybc	Cell parameters from 2000 reflections
*a* = 10.077 (2) Å	θ = 3.2–27.5°
*b* = 8.1235 (16) Å	µ = 2.17 mm^−^^1^
*c* = 17.101 (3) Å	*T* = 293 K
β = 124.86 (3)°	Block, colorless
*V* = 1148.7 (6) Å^3^	0.10 × 0.10 × 0.10 mm
*Z* = 4	

### Data collection

**Table:**

Rigaku R-AXIS RAPID diffractometer	2605 independent reflections
Radiation source: fine-focus sealed tube	1593 reflections with *I* > 2σ(*I*)
Graphite monochromator	*R*_int_ = 0.119
Detector resolution: 10.00 pixels mm^-1^	θ_max_ = 27.5°, θ_min_ = 3.2°
ω scans	*h* = −13→13
Absorption correction: multi-scan (*ABSCOR*; Higashi, 1995)	*k* = −10→10
*T*_min_ = 0.813, *T*_max_ = 0.813	*l* = −22→22
10467 measured reflections	

### Refinement

**Table:**

Refinement on *F*^2^	Primary atom site location: structure-invariant direct methods
Least-squares matrix: full	Secondary atom site location: difference Fourier map
*R*[*F*^2^ > 2σ(*F*^2^)] = 0.062	Hydrogen site location: inferred from neighbouring sites
*wR*(*F*^2^) = 0.142	H atoms treated by a mixture of independent and constrained refinement
*S* = 1.01	*w* = 1/[σ^2^(*F*_o_^2^) + (0.057*P*)^2^] where *P* = (*F*_o_^2^ + 2*F*_c_^2^)/3
2605 reflections	(Δ/σ)_max_ < 0.001
168 parameters	Δρ_max_ = 0.61 e Å^−^^3^
5 restraints	Δρ_min_ = −0.71 e Å^−^^3^

### Special details

**Table:**

Geometry. All e.s.d.'s (except the e.s.d. in the dihedral angle between two l.s. planes) are estimated using the full covariance matrix. The cell e.s.d.'s are taken into account individually in the estimation of e.s.d.'s in distances, angles and torsion angles; correlations between e.s.d.'s in cell parameters are only used when they are defined by crystal symmetry. An approximate (isotropic) treatment of cell e.s.d.'s is used for estimating e.s.d.'s involving l.s. planes.
Refinement. Refinement of *F*^2^ against ALL reflections. The weighted *R*-factor *wR* and goodness of fit *S* are based on *F*^2^, conventional *R*-factors *R* are based on *F*, with *F* set to zero for negative *F*^2^. The threshold expression of *F*^2^ > σ(*F*^2^) is used only for calculating *R*-factors(gt) *etc*. and is not relevant to the choice of reflections for refinement. *R*-factors based on *F*^2^ are statistically about twice as large as those based on *F*, and *R*- factors based on ALL data will be even larger.

### Fractional atomic coordinates and isotropic or equivalent isotropic displacement parameters (Å^2^)

**Table:**

	*x*	*y*	*z*	*U*_iso_*/*U*_eq_	
Zn1	0.44202 (7)	0.83143 (7)	0.20225 (4)	0.0346 (2)	
O1	0.6292 (4)	0.8216 (4)	0.3472 (2)	0.0380 (9)	
O2	0.5778 (4)	1.0836 (4)	0.3099 (2)	0.0389 (9)	
O4	1.1259 (4)	1.3677 (4)	0.7066 (2)	0.0438 (10)	
O5	0.9191 (5)	1.4792 (4)	0.5739 (3)	0.0589 (13)	
O1W	0.3262 (4)	0.9960 (4)	0.1007 (3)	0.0475 (11)	
H1A	0.334 (6)	1.097 (3)	0.110 (3)	0.057*	
H1B	0.244 (4)	0.976 (5)	0.047 (2)	0.057*	
C1	0.6580 (5)	0.9708 (5)	0.3707 (3)	0.0294 (12)	
C2	0.7857 (5)	1.0133 (5)	0.4697 (3)	0.0297 (11)	
O3	0.8150 (4)	1.1781 (3)	0.4901 (2)	0.0329 (8)	
C3	0.9431 (6)	1.1887 (6)	0.5841 (3)	0.0334 (12)	
C5	0.9904 (6)	1.0374 (6)	0.6215 (4)	0.0401 (14)	
H5	1.0737	1.0123	0.6841	0.048*	
C4	0.8892 (6)	0.9213 (6)	0.5474 (3)	0.0365 (13)	
H4	0.8937	0.8071	0.5519	0.044*	
C6	0.9997 (6)	1.3588 (6)	0.6231 (4)	0.0382 (13)	
O7	0.6257 (4)	0.8255 (5)	0.1789 (3)	0.0463 (10)	
H7	0.708 (5)	0.785 (7)	0.224 (3)	0.056*	
C7	0.5999 (8)	0.8006 (8)	0.0889 (4)	0.066 (2)	
H7A	0.5788	0.6861	0.0722	0.080*	
H7B	0.6945	0.8338	0.0923	0.080*	
H7C	0.5089	0.8649	0.0414	0.080*	
O8	0.2706 (5)	0.8407 (4)	0.2328 (3)	0.0519 (10)	
H8	0.225 (6)	0.931 (4)	0.214 (4)	0.062*	
C8	0.2859 (9)	0.7722 (8)	0.3147 (5)	0.0660 (19)	
H8A	0.3299	0.6631	0.3258	0.079*	
H8B	0.1814	0.7675	0.3038	0.079*	
H8C	0.3566	0.8399	0.3692	0.079*	

### Atomic displacement parameters (Å^2^)

**Table:**

	*U*^11^	*U*^22^	*U*^33^	*U*^12^	*U*^13^	*U*^23^
Zn1	0.0342 (4)	0.0245 (3)	0.0305 (3)	0.0015 (2)	0.0098 (3)	0.0008 (3)
O1	0.043 (2)	0.0227 (16)	0.0330 (18)	−0.0025 (15)	0.0124 (16)	−0.0055 (15)
O2	0.040 (2)	0.0242 (17)	0.0311 (19)	0.0018 (15)	0.0075 (17)	0.0067 (15)
O4	0.037 (2)	0.0347 (19)	0.0316 (19)	0.0007 (16)	0.0034 (16)	−0.0017 (16)
O5	0.055 (2)	0.0290 (19)	0.042 (2)	0.0009 (18)	−0.0027 (19)	−0.0061 (18)
O1W	0.050 (2)	0.0228 (17)	0.0315 (19)	−0.0047 (17)	0.0005 (17)	−0.0004 (16)
C1	0.031 (3)	0.020 (2)	0.031 (3)	−0.0002 (19)	0.015 (2)	0.000 (2)
C2	0.029 (3)	0.023 (2)	0.030 (3)	−0.003 (2)	0.012 (2)	−0.009 (2)
O3	0.0324 (18)	0.0215 (15)	0.0272 (16)	−0.0021 (14)	0.0068 (14)	−0.0041 (14)
C3	0.028 (2)	0.032 (3)	0.025 (2)	−0.004 (2)	0.006 (2)	−0.002 (2)
C5	0.033 (3)	0.036 (3)	0.032 (3)	0.000 (2)	0.007 (2)	0.004 (2)
C4	0.036 (3)	0.025 (2)	0.035 (3)	−0.001 (2)	0.013 (2)	0.000 (2)
C6	0.036 (3)	0.034 (3)	0.033 (3)	−0.003 (2)	0.012 (2)	−0.006 (2)
O7	0.041 (2)	0.051 (2)	0.038 (2)	0.0064 (18)	0.0175 (18)	0.0070 (19)
C7	0.071 (5)	0.081 (5)	0.047 (4)	0.019 (4)	0.033 (3)	0.010 (3)
O8	0.048 (2)	0.041 (2)	0.067 (3)	0.0144 (18)	0.033 (2)	0.019 (2)
C8	0.084 (5)	0.054 (4)	0.071 (5)	0.008 (4)	0.051 (4)	0.018 (4)

### Geometric parameters (Å, º)

**Table:**

Zn1—O1W	1.962 (3)	O3—C3	1.372 (5)
Zn1—O2^i^	2.022 (3)	C3—C5	1.341 (6)
Zn1—O8	2.072 (4)	C3—C6	1.499 (6)
Zn1—O1	2.090 (4)	C5—C4	1.435 (6)
Zn1—O7	2.105 (4)	C5—H5	0.9300
Zn1—O2	2.565 (3)	C4—H4	0.9300
O1—C1	1.257 (5)	O7—C7	1.421 (7)
O2—C1	1.270 (5)	O7—H7	0.817 (19)
O2—Zn1^ii^	2.022 (3)	C7—H7A	0.9600
O4—C6	1.259 (5)	C7—H7B	0.9600
O5—C6	1.243 (6)	C7—H7C	0.9600
O1W—H1A	0.828 (19)	O8—C8	1.430 (7)
O1W—H1B	0.828 (19)	O8—H8	0.82 (2)
C1—C2	1.467 (6)	C8—H8A	0.9600
C2—C4	1.352 (6)	C8—H8B	0.9600
C2—O3	1.372 (5)	C8—H8C	0.9600
			
O1W—Zn1—O2^i^	127.86 (14)	C5—C3—C6	133.7 (4)
O1W—Zn1—O8	92.21 (17)	O3—C3—C6	116.3 (4)
O2^i^—Zn1—O8	90.81 (15)	C3—C5—C4	107.5 (4)
O1W—Zn1—O1	138.87 (13)	C3—C5—H5	126.2
O2^i^—Zn1—O1	93.05 (12)	C4—C5—H5	126.2
O8—Zn1—O1	91.10 (16)	C2—C4—C5	105.3 (4)
O1W—Zn1—O7	89.45 (17)	C2—C4—H4	127.3
O2^i^—Zn1—O7	90.23 (15)	C5—C4—H4	127.3
O8—Zn1—O7	176.90 (15)	O5—C6—O4	124.6 (4)
O1—Zn1—O7	85.93 (15)	O5—C6—C3	119.3 (4)
O1W—Zn1—O2	83.95 (13)	O4—C6—C3	116.0 (4)
O2^i^—Zn1—O2	148.19 (7)	C7—O7—Zn1	124.9 (4)
O8—Zn1—O2	88.27 (15)	C7—O7—H7	116 (4)
O1—Zn1—O2	55.19 (11)	Zn1—O7—H7	111 (4)
O7—Zn1—O2	89.31 (13)	O7—C7—H7A	109.5
C1—O1—Zn1	103.2 (3)	O7—C7—H7B	109.5
C1—O2—Zn1^ii^	141.4 (3)	H7A—C7—H7B	109.5
C1—O2—Zn1	80.8 (3)	O7—C7—H7C	109.5
Zn1^ii^—O2—Zn1	137.77 (14)	H7A—C7—H7C	109.5
Zn1—O1W—H1A	124 (3)	H7B—C7—H7C	109.5
Zn1—O1W—H1B	124 (3)	C8—O8—Zn1	126.4 (4)
H1A—O1W—H1B	110 (3)	C8—O8—H8	117 (5)
O1—C1—O2	120.8 (4)	Zn1—O8—H8	107 (4)
O1—C1—C2	119.0 (4)	O8—C8—H8A	109.5
O2—C1—C2	120.2 (4)	O8—C8—H8B	109.5
C4—C2—O3	110.9 (4)	H8A—C8—H8B	109.5
C4—C2—C1	132.8 (4)	O8—C8—H8C	109.5
O3—C2—C1	116.3 (4)	H8A—C8—H8C	109.5
C3—O3—C2	106.3 (3)	H8B—C8—H8C	109.5
C5—C3—O3	110.0 (4)		
			
O1W—Zn1—O1—C1	7.3 (5)	O1—C1—C2—O3	177.9 (4)
O2^i^—Zn1—O1—C1	−178.2 (3)	O2—C1—C2—O3	−2.3 (7)
O8—Zn1—O1—C1	−87.3 (3)	C4—C2—O3—C3	0.7 (6)
O7—Zn1—O1—C1	91.8 (3)	C1—C2—O3—C3	−176.8 (4)
O2—Zn1—O1—C1	−0.2 (3)	C2—O3—C3—C5	−1.1 (6)
O1W—Zn1—O2—C1	−174.9 (3)	C2—O3—C3—C6	179.2 (4)
O2^i^—Zn1—O2—C1	4.0 (3)	O3—C3—C5—C4	1.0 (6)
O8—Zn1—O2—C1	92.7 (3)	C6—C3—C5—C4	−179.3 (6)
O1—Zn1—O2—C1	0.2 (3)	O3—C2—C4—C5	−0.1 (6)
O7—Zn1—O2—C1	−85.3 (3)	C1—C2—C4—C5	176.8 (5)
O1W—Zn1—O2—Zn1^ii^	5.4 (3)	C3—C5—C4—C2	−0.5 (6)
O2^i^—Zn1—O2—Zn1^ii^	−175.7 (3)	C5—C3—C6—O5	−171.4 (6)
O8—Zn1—O2—Zn1^ii^	−87.0 (3)	O3—C3—C6—O5	8.3 (8)
O1—Zn1—O2—Zn1^ii^	−179.5 (3)	C5—C3—C6—O4	6.4 (10)
O7—Zn1—O2—Zn1^ii^	95.0 (3)	O3—C3—C6—O4	−174.0 (5)
Zn1—O1—C1—O2	0.4 (6)	O1W—Zn1—O7—C7	−52.1 (4)
Zn1—O1—C1—C2	−179.9 (4)	O2^i^—Zn1—O7—C7	75.8 (4)
Zn1^ii^—O2—C1—O1	179.4 (4)	O1—Zn1—O7—C7	168.8 (4)
Zn1—O2—C1—O1	−0.3 (5)	O2—Zn1—O7—C7	−136.1 (4)
Zn1^ii^—O2—C1—C2	−0.4 (8)	O1W—Zn1—O8—C8	−167.6 (5)
Zn1—O2—C1—C2	179.9 (5)	O2^i^—Zn1—O8—C8	64.5 (5)
O1—C1—C2—C4	1.1 (9)	O1—Zn1—O8—C8	−28.6 (5)
O2—C1—C2—C4	−179.1 (5)	O2—Zn1—O8—C8	−83.7 (5)

Symmetry codes: (i) −*x*+1, *y*−1/2, −*z*+1/2; (ii) −*x*+1, *y*+1/2, −*z*+1/2.

### Hydrogen-bond geometry (Å, º)

**Table:**

*D*—H···*A*	*D*—H	H···*A*	*D*···*A*	*D*—H···*A*
O1*W*—H1*A*···O1^ii^	0.83 (2)	1.92 (2)	2.745 (5)	171 (5)
O1*W*—H1*B*···O5^i^	0.83 (2)	1.76 (2)	2.571 (5)	165 (5)
O7—H7···O4^iii^	0.82 (2)	1.86 (3)	2.639 (5)	158 (6)
O8—H8···O4^iv^	0.82 (2)	1.88 (3)	2.682 (5)	163 (6)

Symmetry codes: (i) −*x*+1, *y*−1/2, −*z*+1/2; (ii) −*x*+1, *y*+1/2, −*z*+1/2; (iii) −*x*+2, −*y*+2, −*z*+1; (iv) *x*−1, −*y*+5/2, *z*−1/2.
